# Comparison of Sputum-Culture Conversion for *Mycobacterium bovis* and *M. tuberculosis*

**DOI:** 10.3201/eid2303.161916

**Published:** 2017-03

**Authors:** Colleen Scott, Joseph S. Cavanaugh, Benjamin J. Silk, Julia Ershova, Gerald H. Mazurek, Philip A. LoBue, Patrick K. Moonan

**Affiliations:** Centers for Disease Control and Prevention, Atlanta, Georgia, USA

**Keywords:** Mycobacterium bovis, Mycobacterium tuberculosis, bacteria, tuberculosis and other mycobacteria, tuberculosis, disease, sputum, sputum-culture conversion

## Abstract

Current US guidelines recommend longer treatment for tuberculosis (TB) caused by pyrazinamide-resistant organisms (e.g., *Mycobacterium bovis*) than for *M. tuberculosis* TB. We compared treatment response times for patients with *M. bovis* TB and *M. tuberculosis* TB reported in the United States during 2006–2013. We included culture-positive, pulmonary TB patients with genotyping results who received standard 4-drug treatment at the time of diagnosis. Time to sputum-culture conversion was defined as time between treatment start date and date of first consistently culture-negative sputum. We analyzed 297 case-patients with *M. bovis* TB and 30,848 case-patients with *M. tuberculosis* TB. After 2 months of treatment, 71% of *M. bovis* and 65% of *M. tuberculosis* TB patients showed conversion of sputum cultures to negative. Likelihood of culture conversion was higher for *M. bovis* than for *M. tuberculosis*, even after controlling for treatment administration type, sex, and a composite indicator of bacillary burden.

The *Mycobacterium tuberculosis* complex is composed of several genetically related and pathogenic mycobacterial species, including *M. tuberculosis* and *M. bovis* (*1*). Tuberculosis (TB) caused by these species is often clinically indistinguishable, although *M. bovis* has a different epidemiologic profile (*2*–*5*). Despite similarities, there is growing evidence that diversity within the *M. tuberculosis* complex has major immunologic consequences, which may influence treatment response (*6*–*11*). Sputum-culture conversion (i.e., conversion from positive to negative culture result) is considered the principal prognostic indicator for treatment response and is often used as a surrogate endpoint in early-phase randomized clinical trials (*12*,*13*). Studies have demonstrated that sputum-culture conversion differs by *M. tuberculosis* phylogenetic lineage (*14*,*15*).

*M. bovis* is generally considered intrinsically resistant to pyrazinamide, which is considered an essential first-line anti-TB drug. Pyrazinamide is a sterilizing drug that acts synergistically with rifampin to shorten the duration of anti-TB treatment from 9 to 6 months (*16*). In the absence of this benefit, many experts currently recommend extending treatment for TB caused by *M. bovis* (*17*). However, these recommendations are based on expert opinion and lack definitive evidence from laboratory studies or randomized clinical trials (*17*). We evaluated differences in time from treatment initiation to sputum-culture conversion between patients with *M. bovis* TB and *M. tuberculosis* TB given standard first-line anti-TB treatment.

## Methods

We analyzed data from the National Tuberculosis Surveillance System (NTSS) at the Centers for Disease Control and Prevention (Atlanta, GA, USA) and restricted analysis to cases reported during 2006–2013 to permit sufficient time for follow-up reporting of outcome data. *M. tuberculosis* complex isolates were identified by using spoligotyping and multilocus variable number tandem repeat (i.e., mycobacterial interspersed repetitive unit−variable number tandem repeat) genotyping techniques (*3*,*18*).

We used a retrospective cohort study design and included culture-confirmed TB cases with complete genotyping results and pulmonary disease treated with a standard 4-drug regimen (i.e., isoniazid, rifampin, ethambutol, and pyrazinamide) at diagnosis (*19*). Cases with isolates identified as any species other than *M. tuberculosis* or *M. bovis* were excluded; cases with isolates identified as *M. bovis* Bacillus Calmette–Guérin were assumed to be iatrogenic (*20*) and were also excluded. We excluded from analysis any case-patients with organisms initially resistant to rifampin or isoniazid, those infected with *M. tuberculosis* initially resistant to pyrazinamide, those who were dead at time of diagnosis, and those with missing or unreliable culture-conversion data. We used the Pearson χ^2^ test to compare clinical and demographic characteristics of case-patients with *M. bovis* and *M. tuberculosis* TB and the proportion of case-patients who showed conversion of cultures at 2 and 3 months.

Time to sputum-culture conversion was calculated for persons with positive sputum cultures as the number of days from the date treatment started until the date of the first consistently culture-negative sputum. The date of the first consistently culture-negative sputum was defined as the date that a specimen was collected for the first documented negative culture result with no concurrent (i.e., samples collected within 1 week) or subsequent positive cultures. We used a Kaplan-Meier estimator to calculate survival-like function curves for time to sputum-culture conversion for persons with *M. bovis* and *M. tuberculosis* TB.

We censored, at the date recorded for each outcome, patients who died during treatment and those who moved, were lost to follow-up, or otherwise stopped treatment before the expected completion date; we restricted the analysis to the first 90 days of anti-TB treatment. We used Cox proportional hazard modeling to calculate adjusted hazard ratios (aHRs) with 95% CIs of factors associated with time to culture conversion. We tested the proportional hazards assumption by graphing log (–log [survival probability]) versus log (time) for all covariates of interest. We assessed a priori covariates of interest, including sex; treatment administration type (directly observed therapy versus self-administered therapy); reported HIV status (positive, negative, and unknown); sputum smear status (positive, negative, not obtained, and unknown); and cavitary disease found by diagnostic imaging (chest radiography or computed tomography; positive, negative, not done, and unknown) results.

After reviewing smear status and diagnostic imaging results, we created a composite variable for bacillary burden (high, medium, low, and unknown) to avoid collinearity. We categorized cases with confirmed cavitary disease identified by imaging and positive sputum smear as high bacillary burden, cases with either confirmed cavitary disease or positive sputum smear as medium bacillary burden, cases with no cavitary disease and negative sputum smears as low bacillary burden, and cases without any of these indicators as unknown bacillary burden. In the time-to-event analysis, we excluded patients who had unknown bacillary burden. Analysis was conducted by using SAS version 9.3 (SAS Institute, Cary, NC, USA).

## Results

**Figure 1:**
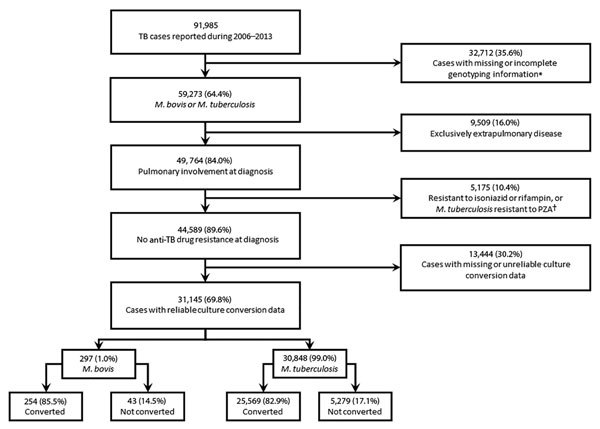
Selection of tuberculosis cases for analysis of sputum-culture conversion, United States, 2006–2013. Analysis included cases of culture-positive disease. A total of 61% of case-patients with *M. bovis* TB and 63% of case-patients with *M. tuberculosis* TB met analytic requirements for inclusion. PZA, pyrazinamide; TB, tuberculosis. *Mycobacterium tuberculosis* with pyrazinamide resistance (n = 757).

A total of 91,985 TB cases during 2006–2013 were available for analysis (Figure 1). Approximately two thirds (64.4%) of these cases had complete genotyping results and were identified as either *M. tuberculosis* TB or *M. bovis* TB. All cases of TB with initial resistance to isoniazid or rifampin (n = 4,418) and *M. tuberculosis* TB cases with any initial resistance to pyrazinamide (n = 757) were excluded. The final dataset for analysis included 297 cases of *M. bovis* TB and 30,848 cases of *M. tuberculosis* TB. All covariates met the proportional hazards assumption except for HIV status, which was excluded from further analysis because of sparse data.

The age distributions of patients given a diagnosis of *M. bovis* TB and patients given a diagnosis of *M. tuberculosis* TB were similar (Table 1). A greater proportion of patients with *M. bovis* TB were female (123/297 [41.1%]) than patients with *M. tuberculosis* TB (10,536/30,848 [34.2%]; p = 0.01). Patients with *M. bovis* TB were most often born outside the United States (86.2%), and most self-identified as Hispanic (93.2%). In comparison, just over half (59.5%) of *M. tuberculosis* TB patients were born outside the United States; 30.3% self-identified as Hispanic (p<0.0001 for both comparisons).

**Table 1:** Characteristics of patients with pulmonary tuberculosis caused by *Mycobacterium bovis* or *Mycobacterium tuberculosis*, United States, 2006–2013*

Variable	Cause of tuberculosis		p value†
	*M. bovis*, n = 297	*M. tuberculosis*, n = 30,848	
Age, y			
0–4	0	18 (0.1)	0.52
5–14	2 (0.7)	202 (0.7)	
15–24	46 (15.5)	3966 (12.9)	
25–44	105 (35.4)	10,553 (34.2)	
45–64	99 (33.3)	10,237 (33.2)	
>65	45 (15.2)	5872 (19.0)	
Median age, y (interquartile range)	43 (29–56)	46 (30–60)	0.12
Sex			
M	174 (58.6)	20,303 (65.8)	0.01
F	123 (41.4)	10,536 (34.2)	
Race/ethnicity			
White, non-Hispanic	9 (3.0)	5205 (16.8)	<0.0001
Native American, non-Hispanic	0	456 (1.5)	
Asian, non-Hispanic	3 (1.0)	7869 (26.0)	
Black, non-Hispanic	8 (2.7)	7578 (25.0)	
Hispanic	276 (93.2)	9177 (30.3)	
Country of birth			
United States	41 (13.8)	12,442 (40.3)	<0.0001
Other‡	256 (86.2)	18,371 (59.5)	
Reported HIV status§			
Positive	17 (5.7)	1851 (6.0)	<0.0001
Negative	166 (55.9)	21,585 (70.0)	
Unknown	114 (38.4)	7412 (24.0)	
Clinical presentation at diagnosis			
Pulmonary disease	193 (65.0)	27,757 (90.0)	<0.0001
Pulmonary and extrapulmonary disease	104 (35.0)	3090 (10.0)	
Computed tomography or other chest imaging findings¶			
Cavitary disease	90/193 (46.6)	12,476/27,757 (45.0)	0.64
Noncavitary disease	103/193 (53.4)	15,281/27,757 (55.0)	
Sputum smear result			
Positive	189 (63.6)	20,744 (67.3)	0.40
Negative	98 (33.0)	9403 (30.5)	
Not obtained	10 (3.4)	689 (2.2)	
Bacillary burden#			
High	84 (28.3)	11,154 (36.2)	0.03
Medium	120 (40.4)	11,445 (37.1)	
Low	85 (28.6)	7734 (25.1)	
Unknown	8 (2.7)	515 (1.7)	
Treatment outcome**			
Completed	235 (83.9)	25,535 (90.5)	0.001
Died	25 (8.9)	1447 (5.1)	
Other††	20 (7.4)	1238 (4.4)	

*Values are no. (%) unless otherwise indicated. Sums of counts across categories for a specific factor might be less than total counts in the column headings because of missing or incomplete data. †Determined by using Pearson χ^2^ test unless otherwise indicated. ‡Restricted to foreign-born persons. §HIV status data are unknown for patients reported for California and Vermont during 2006–2011. Unknown also includes data from other states reporting unknown HIV status for tuberculosis patients. ¶Cavitary status was documented if reports of either conventional radiography or computed tomography indicated its presence. #A bacillary burden composite variable was created by using smear status and radiographic evidence of cavitation. **Outcome data are available for, and restricted to, patients reported during 2006–2013. ††Includes treatment stopped because of adverse events; patients were lost to follow-up, moved, refused treatment; and unknown reasons.

Approximately one third (35.0%) of *M. bovis* TB patients had pulmonary and extrapulmonary involvement at diagnosis compared with 10.0% of *M. tuberculosis* TB patients (p<0.0001). Similar proportions of patients had cavitary chest lesions documented (*M. bovis* TB = 46.6%, *M. tuberculosis* TB = 45.0%; p = 0.64) and positive sputum smear results (*M. bovis* TB = 63.6%, *M. tuberculosis* TB = 67.3%; p = 0.40). Among *M. tuberculosis* TB patients, proportions categorized as high bacillary burden (36.2%) and medium bacillary burden (37.1%) differed significantly from those for *M. bovis* TB patients (28.3% and 40.4%, respectively) (p = 0.03).

**Figure 2:**
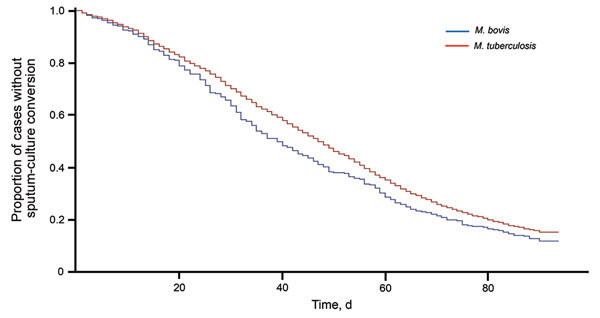
Time to sputum-culture conversion for case-patients with *Mycobacterium bovis* and *M. tuberculosis* TB, United States, 2006–2013. At day 0, a total of 297 persons had culture-positive *M. bovis* TB and 30,848 had culture-positive *M. tuberculosis* TB; at day 20, a total of 239 persons had culture-positive *M. bovis* TB and 25,363 had culture-positive *M. tuberculosis* TB; at day 40, a total of 143 persons had culture-positive *M. bovis* TB and 17,882 had culture-positive *M. tuberculosis* TB; at day 60, a total of 85 persons had culture-positive *M. bovis* TB and 10,853 had culture-positive *M. tuberculosis* TB; and at day 80, a total of 47 persons had culture-positive *M. bovis* TB and 6,084 had culture-positive *M. tuberculosis* TB. TB, tuberculosis.

At 2 months of treatment, 71% of *M. bovis* TB patients and 65% of *M. tuberculosis* TB patients showed conversion of their sputum cultures to negative (p<0.01) (Figure 2). By the end of 3 months of treatment, 86% of *M. bovis* TB patients and 83% of *M. tuberculosis* TB patients showed conversion of their sputum cultures to negative (Figure 2). On the basis of Cox proportional hazards regression modeling, we found that *M. bovis* TB patients had a higher hazard of conversion of sputum cultures to negative (aHR 1.18, 95% CI 1.04–1.33) relative to *M. tuberculosis* TB patients, after controlling for treatment administration type, sex, and the composite indicator for bacillary burden (Table 2). Directly observed therapy (aHR 1.12, 95% CI 1.09–1.15, relative to self-administered therapy) and female sex (aHR 1.15, 95% CI 1.12–1.18) were also found to increase the hazards of sputum-culture conversion. A graded response to bacillary burden was observed (low, aHR 1.68, 95% CI 1.63–1.74; medium, aHR 1.32, 95% CI 1.29–1.36) relative to high bacillary burden.

**Table 2:** Hazard ratios for time to sputum-culture conversion among tuberculosis patients with pulmonary diagnoses, United States, 2006–2013*

Factor	Unadjusted HR (95% CI)	Adjusted HR (95% CI)
Cause of tuberculosis		
* Mycobacterium bovis*	1.18 (1.05–1.34)	1.18 (1.04–1.33)
* M. tuberculosis*	Referent	Referent
Treatment administration type		
Directly observed therapy	1.08 (1.05–1.11)	1.12 (1.09–1.15)
Self-administered	Referent	Referent
Sex		
F	1.17 (1.14–1.20)	1.15 (1.12–1.18)
M	Referent	Referent
Bacillary burden†		
Low	1.61 (1.56–1.66)	1.68 (1.63–1.74)
Medium	1.25 (1.22–1.29)	1.32 (1.29–1.36)
High	Referent	Referent

*HR, hazard ratio. †A bacillary burden composite variable was created by using smear status and radiographic evidence of cavitation. Patients with unknown bacillary burden were excluded from analyses.

## Discussion

For patients given a standard 4-drug regimen in the United States, we found that the hazard of culture conversion over the first 3 months of anti-TB treatment was higher for patients with *M. bovis* TB than for patients with *M. tuberculosis* TB after controlling for treatment administration type, sex, and a composite indicator of bacillary burden. This finding was not documented previously and is especially intriguing because *M. bovis* is inherently resistant to pyrazinamide, a first-line anti-TB drug used during the first 2 months of treatment and credited with reducing relapse rates of the 6-month regimen to levels similar to the 9-month regimen without this drug (*17*). The implications of this inherent resistance are unknown. A systematic review of treatment for *M. bovis* TB concluded that the effect of 6-month versus 9-month treatment durations could not be determined because of a paucity of observational data (*21*). Our findings suggest that TB caused by *M. bovis* might not require a 9-month treatment regimen. However, this suggestion requires additional investigations.

We found that *M. bovis* TB patients had pulmonary and extrapulmonary involvement at diagnosis more frequently than *M. tuberculosis* TB patients, which is consistent with previous reports describing the transmission and epidemiology of *M. bovis* (*2*,*3*,*22*,*23*). However, our analysis showed that *M. bovis* TB patients with pulmonary disease might have been less infectious (i.e., had a lower bacillary burden) than *M. tuberculosis* TB patients. We also found similar proportions of cavitary lesions on chest imaging, which might indicate a similar pathogenicity between organisms when pulmonary disease is present. However, this finding could not be directly studied because NTSS does not collect data on smear grade. Alternatively, differential gene expression, proinflammatory macrophage response, growth in macrophages, and lipid profiles might have roles in explaining variability in bacillary populations between species (*7*–*11*). Further studies assessing differences in bacillary burden, and how anti-TB treatment is affected, will help clarify potential differences in treatment efficacy for *M. tuberculosis* complex species.

The true global burden of *M. bovis* TB is unknown and estimates are imprecise. Populations burdened by endemic zoonotic TB, such as large pastoralist populations who live near livestock and communities with increased consumption of unpasteurized dairy products, are often located where a specific *M. bovis* TB diagnosis is unlikely because extrapulmonary cases are not easily diagnosed and access to molecular technologies (e.g., genotyping) and drug susceptibility testing are not readily available (*24**–**27*). TB treatment strategies are moving toward shorter, more effective anti-TB regimens. If our finding of more rapid time to sputum-culture conversion for *M. bovis* TB could be replicated with more robust randomized clinical trial studies or hollow-fiber models (*25*,*26*), recommendations for treatment might be improved. Modifying these standards could reduce the recommended treatment length for potentially hundreds of thousands patients globally, assuming a conservative 2%–3% *M. bovis* TB prevalence estimate among the 9.6 million cases of TB reported each year (*27*). As research elucidates mycobacterial features and characteristics, individualized treatment regimens might be tailored to specific organisms. However, many potential new regimens include pyrazinamide as an essential drug throughout the entire treatment course (*28*).

This study and its findings are subject to the limitations of the national surveillance data we used. Historically, there has been variation among states in the reporting of HIV testing results (*29*). California, which reports more than half of all *M. bovis* TB cases nationally each year, began reporting HIV test results to the Centers for Disease Control and Prevention in 2011. This reporting limited our ability to analyze any association between HIV and time to sputum-culture conversion for this study. Although there is a standard recommendation for follow-up sputum collection frequency after initiation of TB treatment, clinician and patient variability in implementation is likely. However, because there is no reason to believe that this variation would be implemented differently for *M. bovis* TB patients versus *M. tuberculosis* TB patients, this limitation is not likely to have affected comparison on the basis of species.

We attempted to control for potential variations in treatment by including only patients initially given a standard 4-drug regimen (isoniazid, rifampin, ethambutol, and pyrazinamide daily for 2 months). However, some clinicians might have changed regimens after receiving genotyping results (e.g., *M. bovis*) or drug susceptibility testing results (e.g., pyrazinamide resistance). Thus, some patients with *M. bovis* genotyping results or pyrazinamide resistance might have received a different regimen at some point after the start of treatment. NTSS does not capture information on changes to regimens during the course of treatment or the date when drug susceptibility testing results were received, and we were unable to assess this information directly.

In 2013, time from treatment start date to date of linked genotyping results was a median of 107 days (range 15–365 days). Thus, for 50% of cases, clinicians would have received genotyping results after the event time of this analysis (i.e., 90 days), which would diminish any potential influence on our findings. Moreover, removing pyrazinamide from regimens used to treat *M. bovis* TB cases would have not have affected time to culture conversion; rather, it would have prolonged it.

Concurrent and immunosuppressive conditions, such as diabetes mellitus, end-stage renal disease, and hematologic or reticuloendothelial malignancies, which might influence time to culture conversion, were not routinely collected during our study period. These variables were included as part of routine reporting in 2009 and might be helpful in future studies (*29*). Because the proportion of race/ethnicity differed by bacterial species, and some race/ethnicities have a higher prevalence of concurrent conditions (e.g., diabetes mellitus) that might influence time to culture conversion, we attempted to run a separate hazard model on the basis of race/ethnicity, but this variable did not satisfy the proportional hazards assumption.

When we restricted analysis to only Hispanic persons, *M. bovis* TB patients had similar hazards of converting sputum cultures to negative (HR 1.20, 95% CI 1.10–1.35) as in our adjusted model (Table 2). This finding suggests that self-identifying as Hispanic had no effect on our main finding that *M. bovis* TB cases had a higher hazards of converting sputum cultures to negative relative to *M. tuberculosis* TB cases. Although we attempted to control for bacillary burden by using a novel composite variable, smear grade is not reported in NTSS, and our estimates on the effect of bacillary burden might be inaccurate.

Our findings should be interpreted with caution. Although we found earlier culture conversion for *M. bovis* TB patients than for *M. tuberculosis* TB patients, a larger proportion of *M. bovis* TB patients (8.9% vs. 5.1%) died during treatment. This finding suggests that earlier culture conversion does not necessarily lead to better clinical outcomes. Further laboratory studies should be conducted to better monitor and assess the time to sputum-culture conversion and clinical outcomes between these 2 *M. tuberculosis* complex species. If similar results are observed, further randomized clinical trials for treatment duration (and possibly treatment regimen) might be warranted.
